# Resilience and stability of the CF- intestinal and respiratory microbiome during nutritional and exercise intervention

**DOI:** 10.1186/s12866-023-02788-y

**Published:** 2023-02-21

**Authors:** Rebecca L. Knoll, Víctor Hugo Jarquín-Díaz, Jonas Klopp, Alissa Kemper, Katja Hilbert, Barlo Hillen, Daniel Pfirrmann, Perikles Simon, Viola Bähner, Oliver Nitsche, Stephan Gehring, Lajos Markó, Sofia K. Forslund, Krystyna Poplawska

**Affiliations:** 1 Pediatric Pulmonology, Allergology and Cystic Fibrosis, Children’s Hospital, University Medical Center of the Johannes Gutenberg-University Mainz, Germany; Langenbeckstraße 1, 55131 Mainz, Germany; 2 Pediatric Immunology and Infectiology, Children’s Hospital, University Medical Center of the Johannes Gutenberg-University Mainz, Germany; Langenbeckstraße 1, 55131 Mainz, Germany; 3 Experimental and Clinical Research Center, a cooperation between the Max Delbrück Center for Molecular Medicine in the Helmholtz Association and Charité Universitätsmedizin Berlin, Berlin, Germany; 4 Charité – Universitätsmedizin Berlin, corporate member of Freie Universität Berlin and Humboldt-Universität Zu Berlin, Experimental and Clinical Research Center, Lindenberger Weg 80, 13125 Berlin, Germany; 5 Max Delbrück Center for Molecular Medicine in the Helmholtz Association (MDC), Berlin, Germany; 6 Department of Sports Medicine, Prevention, and Rehabilitation, Faculty of Social Science, Media and Sports, Johannes Gutenberg-University Mainz, Germany; Albert-Schweitzer Str.22, 55128 Mainz, Germany; 7 Structural and Computational Biology Unit, European Molecular Biology Laboratory, Heidelberg, Meyerhofstraße 1, 69117 Heidelberg, Germany; 8 DZHK (German Centre for Cardiovascular Research), Partner Site Berlin, Berlin, Germany

**Keywords:** Cystic fibrosis, Microbiome, Gut-lung axis, Physical exercise, Nutrition, Antibiotics, Intervention

## Abstract

**Background:**

Impaired respiratory and intestinal microbiome composition is linked to cystic fibrosis lung disease severity. In people with cystic fibrosis (pwCF), regular exercise is recommended to delay disease progression and preserve a stable lung function. An optimal nutritional status is vital for best clinical outcomes. Our study investigated whether regular and monitored exercise and nutritional support promotes CF microbiome health.

**Methods:**

A personalized nutrition and exercise program promoted nutritional intake and physical fitness in 18 pwCF for 12 months. Throughout the study, patients performed strength and endurance training monitored by a sports scientist via an internet platform. After three months, food supplementation with *Lactobacillus rhamnosus* LGG was introduced. Nutritional status and physical fitness were assessed before the study started, after three and nine months. Sputum and stool were collected, and microbial composition was analyzed by 16S rRNA gene sequencing.

**Results:**

Sputum and stool microbiome composition remained stable and highly specific to each patient during the study period. Disease-associated pathogens dominated sputum composition. Lung disease severity and recent antibiotic treatment had the highest impact on taxonomic composition in stool and sputum microbiome. Strikingly, the long-term antibiotic treatment burden had only a minor influence.

**Conclusion:**

Despite the exercise and nutritional intervention, respiratory and intestinal microbiomes proved to be resilient. Dominant pathogens drove the composition and functionality of the microbiome. Further studies are required to understand which therapy could destabilize the dominant disease-associated microbial composition of pwCF.

**Supplementary Information:**

The online version contains supplementary material available at 10.1186/s12866-023-02788-y.

## Background

Cystic fibrosis (CF) is an autosomal-recessive genetic disorder. Due to mutations in the *cystic fibrosis transmembrane conductance regulator* (*CFTR)* gene, a defective or absent *CFTR* channel leads to an impaired chloride-sodium balance at cell surfaces, followed by a thickening of mucus in many organs. CF is a multi-organ disease affecting the lungs, the gastrointestinal and the reproductive organs [1].

In the lungs, impaired airway clearance leads to the accumulation of respiratory secretions and subsequent inflammation. This environment promotes the growth of pathogenic microbes such as *Pseudomonas aeruginosa*, *Staphylococcus aureus* or *Stenotrophomonas maltophilia* [2]. Frequent administration of antibiotics to treat progressive, chronic lung infections, generates a significant selective pressure on the lung and the gut microbiome and aggravates microbial dysbiosis [3]. However, even before antibiotic treatment, respiratory [4, 5] and intestinal microbiome dysbiosis is observed in infants and dysfunction in the *CFTR* channel is the primary associated cause [3, 6–9].

Abnormal microbiome colonization in the gastrointestinal and respiratory system amplifies undernutrition, growth failure, systemic inflammation and may result in pulmonary infections [10]. Reduced respiratory microbiome diversity and richness and increased dominance of CF-pathogens have been identified to correlate with lung function decline and disease deterioration [11, 12]. Gut dysbiosis is linked to intestinal inflammation and subsequent barrier impairment [9]. Most importantly, gut dysbiosis facilitates airway disease in CF, as distinct intestinal microbial composition patterns are associated with pulmonary infections already in the first year of life [7] Thus, the gut microbiome has direct implications for the course of lung disease in CF. This ability of the gut microbiome to influence lung physiology and pathology and vice versa is referred to as the gut-lung axis [13]. The connection of the gut-lung axis with CF health outcomes underlines the enormous need to decipher strategies to promote intestinal and respiratory microbiome health in CF [13].

Diet and exercise represent two potential means to influence microbiome composition in CF. Diet impacts microbiome composition [14, 15]. Diet high in fiber or in fermented foods might influence microbiome-mediated immune responses [16]. In order to improve health outcomes in CF, modulating the microbiome via diet or probiotic supplementation is a very appealing approach. In pwCF, probiotic intake has been associated with fewer pulmonary exacerbations and may decrease CF-related gastrointestinal inflammation [9, 17–19]. However, the evidence of probiotic benefits in CF is still limited. Probiotic studies are needed to provide further insights into the pivotal role of the gut-lung axis in CF [9].

Multiple studies linked physical exercise with gut microbiome modulation (reviewed in [20]). The relation between exercise and gut microbiota composition seems to be bidirectional [21]. Regular physical exercise modulates the gut microbiota composition, and on the other hand, gut microbiota composition impacts the physical exercise capacity of the host [21]. The impact of physical exercise on the respiratory microbiome has not been studied so far.

We offered a personalized nutrition and exercise program to pwCF for the duration of one year. We hypothesized that lifestyle changes, including increased physical activity, nutritional improvements and probiotic supplementation promotes intestinal and respiratory microbiome composition, thus delays disease progression in pwCF.

## Results

### Study population characteristics and evolution of clinical parameters

Out of 18 recruited pwCF, 15 concluded the study. Cohort characteristics are displayed in Table 1. Primary goal of the exercise intervention was to increase exercise participation in pwCF. This goal was met, as participants increased their training minutes per week about 42% due to the offered program [22].

**Table 1:** Baseline characteristics and evolution of clinical parameters throughout the study period

Baseline characteristics		Evolution of clinical parameters				
	**Baseline value**^Φ^		**Visit 1**^Φ^	**Visit 2**^Φ^	**Visit 3**^Φ^	*F* = *, p* = *
Male: female	10/18 (56): 8/18 (44)	*N*	18	17	15	
Age (years)	29.5 ± 20.5	*ppFEV*_*1*_	73.6 ± 25.6	73.3 ± 30.5	71.5 ± 35.1	2.27, 0.12
∆F508 homozygous	6 (33)	*ppFVC*	87.9 ± 14.2	86.2 ± 19.6	86.4 ± 23.6	0.76, 0.48
∆F508 heterozygous	8 (44)	*BMI (kg/m*^*2*^*)*	22.0 ± 2.5	22.0 ± 2.6	22.4 ± 3.0	1.01, 0.36
Mutation others	4 (22)	*FFM (kg)*	42^∇^ ± 16.7	43.5^∇^ ± 16.7	50.2^∇^ ± 19.0	3.49, **0.05**
		*VO*_*2*_*peak (ml/min/kg)*	28.7^◊^ ± 10.4	31.5^◊^ ± 9.0	27.9^◊^ ± 9.4	0.24, 0.79
﻿Pancreatic insufficiency	17 (94)	^Φ^ N (%) or Median ± IQR * ANOVA with repeated measures ^∇^ Visit 1&2 *N* = 17, Visit 3 *N* = 12 ^◊^ Visit 1 *N* = 17, Visit 2 *N* = 15, Visit 3 *N* = 11				
CF-related diabetes	7 (39)					
CF-related liver disease	9 (56)					

Clinical assessments and microbiome characterization were performed at baseline (Visit 1), after 3 months (Visit 2) and after 12 months (Visit 3). Cardiopulmonary exercise testing (CPET) was performed to assess cardiorespiratory fitness. VO_2_peak measured during CPET remained stable, while a decline of lung function was observed in the same period. At Visit 2 & 3 nutritional counseling on the basis of 3-days weighing food protocols was offered to the participants. Fat intake was in 51% and protein intake in 28% of the assessments within the nutritional recommendations for pwCF (35%-45% of total caloric intake from fat and > 20% from protein [23]). A change in this macronutrient intake and eating habits did not occur throughout the study period (Supplementary Table 1). To assess body composition bioelectrical impedance analysis was performed to measure fat free mass (FFM) as a proxy for muscle mass. During the exercise intervention BMI remained stable, FFM increased in contrast.

### Microbial communities remain stable during the study period

To determine whether the intervention had an impact on stool and sputum microbiome, we compared overall alpha diversity metrics (diversity, richness, evenness and dominance) between visits. We observed no difference in alpha diversity over time (*p* > 0.05) (Supplementary Fig. 1).

**Fig. 1:**
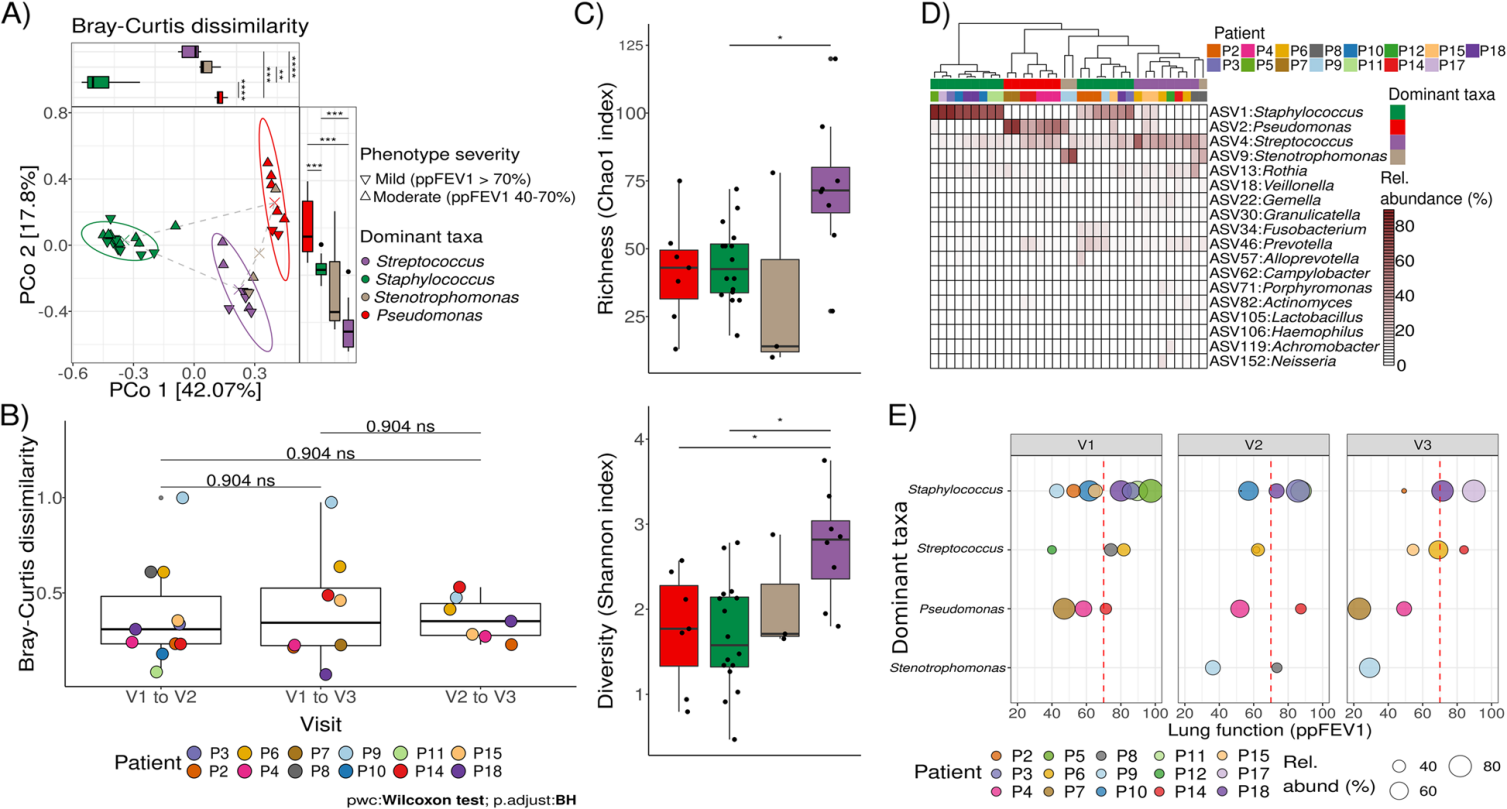
**A** Composition of sputum microbiome was highly explained by dominant taxa and severity of the disease between patients. Principal Coordinate Analysis (PCoA) based on Bray–Curtis dissimilarity between samples. Points are colored by the dominant taxa detected and the shape indicates the phenotype severity based on ppFEV_1_. Crosses indicate the centroid of each cluster and the dashed lines the mean distances between clusters. Ellipses represent the 95% confidence interval of intra-cluster variability of sample distance to the centroid. Distances on PCo1 and PCo2 between groups based on dominant taxa were compared by Mann–Whitney U tests with Benjamini–Hochberg correction for multiple testing (Signif. codes: 0 ‘***’ 0.001 ‘**’ 0.01 ‘*’ 0.05 ‘.’ 0.1). Cluster of *Staphylococcus* dominated samples were significantly different to clusters with *Pseudomonas* and *Streptococcus* dominated microbiomes on PCo1 and PCo2. **B** Changes in sputum microbiome composition within patients between visits. A shift of dominance between *Staphylococcus* to *Stenotrophomonas* was linked to a high Bray–Curtis dissimilarity between visits for patient 9 (P9). For panel B and E each color represents samples from the same patient. **C** Association of dominant genera to alpha-diversity measures in sputum microbiomes. **D** Heatmap of the most abundant genera (> 0.5% mean relative abundance) for each cluster of dominant taxa observed on panel A). Dendrogram indicates Ward clustering of samples. **E** Relationship between dominant taxa in sputum and lung function. Lung function is represented by ppFEV1 at each visit, points are colored by patient and the size represents the relative abundance in percentage at the sample. Dashed read line indicates the threshold of 70% ppFEV1 to separate moderate (< 70%) from mild (> 70%) severity

We confirmed stability of the microbiome composition over the three visits, measured as inter-sample Bray–Curtis dissimilarity and visualized using principal coordinates analysis (PCoA)) (Fig. 1 A-B, Supplementary Fig. 3).

#### Dominant taxa and disease severity are the main drivers of community structure

CF severity significantly impacted clustering of sputum or stool microbial compositions, explaining 8% and 7% of variance, respectively (Fig. 1A displays sputum clustering, Supplementary Fig. 3A displays stool clustering, statistical results of PERMANOVA are displayed in Table 2). Highest explanation of variance was explained by dominant taxa, dominant taxa were highly related to changes in microbial compositions and explained 55% and 32% of variance in sputum and stool, respectively (Table 2).

**Table 2:** PERMANOVA for microbial taxa, functional gene and metabolic pathways

		***Microbial taxa***			***Functional genes***			***Metabolic pathways***		
**Parameter**	**DF**	***F***	**R**^**2**^	**FDR**	***F***	**R**^**2**^	**FDR**	***F***	**R**^**2**^	**FDR**
***Sputum***										
Severity	1	6.5	0.08	0.0030**	4.3	0.05	0.0300*	3.6	0.05	0.0660
Dominant taxa	3	15.6	0.55	0.0030**	15.2	0.56	0.0060**	9.8	0.45	0.0060**
Sex	1	2.2	0.03	0.1080	2.8	0.03	0.0920	3.4	0.05	0.0660
Age	1	1.2	0.01	0.3276	1.3	0.02	0.3108	1.2	0.02	0.3516
Visit	2	0.7	0.02	0.7540	0.4	0.01	0.9040	0.3	0.01	0.9270
BMI	1	1.8	0.02	0.1980	2.4	0.03	0.1170	2.6	0.04	0.0825
Residuals	24	-	0.29	-	-	0.30	-	-	0.37	-
Total	33	-	1.00	-	-	1.00	-	-	1.00	-
***Stool***										
Severity	1	3.5	0.07	0.0020**	1.4	0.03	0.5000	1.0	0.03	0.6980
Dominant taxa	9	1.8	0.32	0.0020**	1.4	0.30	0.5000	1.4	0.31	0.5070
Sex	1	2.7	0.05	0.0020**	0.4	0.01	0.8712	0.5	0.01	0.7752
Age	1	1.3	0.03	0.1848	0.5	0.01	0.8712	0.5	0.01	0.7752
Visit	2	0.6	0.02	0.9990	0.3	0.02	0.9430	0.2	0.01	0.9820
BMI	1	1.9	0.04	0.0090**	2.9	0.07	0.3720	2.7	0.07	0.9820
Residuals	23	-	0.46	-	-	0.56	-	-	0.56	-
Total	38	-	1.00	-	-	1.00	-	-	1.00	-

Displayed are PERMANOVA results on microbial taxa, functional gene and metabolic pathways composition in CF- sputum and stool samples in relation to disease severity, dominant taxa, sex, age, BMI and visit along the study. DF = degrees of freedom; *F* = F value by permutation and stratified by patient, *p*-values based on 999 permutations, FDR-corrected

–-

Signif. codes: 0 ‘***’ 0.001 ‘**’ 0.01 ‘*’ 0.05 ‘.’ 0.1 ‘’ 1

In sputum, *Pseudomonas, Staphylococcus*, *Stenotrophomonas* and *Streptococcus* generated four clusters (Fig. 1A). The intra-cluster variability was measured as the distance to each cluster centroid, the cluster of *Streptococcus* dominated samples has less variability among them than those clusters dominated by *Pseudomonas* or *Staphylococcus* (Supplementary Fig. 2). For stool just *Bacteroides* seemed to drive the clustering and separated samples from the rest (Supplementary Fig. 3A). BMI, sex, and age did not show a significant effect on microbial composition in sputum, contrary to the idea of the individuality of bacterial composition (Table 2). Visit, as a proxy for the intervention, did not explain changes in microbial composition. Disease severity and dominant taxa explained the composition of predicted functional genes for sputum, but not for stool.

#### The relative abundance of dominant taxa in sputum as an indicator of poor lung function

Considering the significant impact of dominant taxa in the composition of sputum, we explored whether the relative abundance of *Pseudomonas*, *Staphylococcus*, *Streptococcus* and *Stenotrophomonas* were linked to the lung function parameters. During the three visits we observed that the dominance of *Pseudomonas* was linked to ppFEV_1_ values below 70%, thus related to moderate-severe lung disease. On the contrary, even a high relative abundance of *Staphylococcus* occurred in patients with ppFEV_1_ values above 70% (Fig. 1E). *Streptococcus* dominated lung microbiome in individuals with better lung function, also displaying higher genus richness and diversity (Fig. 1C-E). A strong correlation of lung function parameters and alpha diversity metrics, as in previous studies [11, 24] was not reproducible (Supplementary Figs. 4 and 5 and Supplementary Tables 4 and 5).

### Sport intervention lacks impact on microbial composition

We tested whether lung function and/or training parameters including time and frequency could explain BC-dissimilarity as a proxy for changes in individual microbial composition (Supplementary Fig. 6 A and B, Supplementary Tables 2 and 3).

Lung function parameters, but not training frequency or time, significantly explained changes in stool microbiome (ppFEV1:LRT, df = 1, *P* < 0.001, ppFVC: LRT, df = 1, *P* < 0.001). For sputum, neither training nor lung function parameters were significant explanatory variables of microbial changes (Supplementary Table 3), suggesting the stability and lower-complexity of sputum compared to stool. Sputum microbial changes only occurred in a small subset of patients, in which composition was strongly and significantly related to the dominant taxa present (Table 2).

### Association of microbial features to clinical and nutritional characteristics (Fig. 2)

**Fig. 2:**
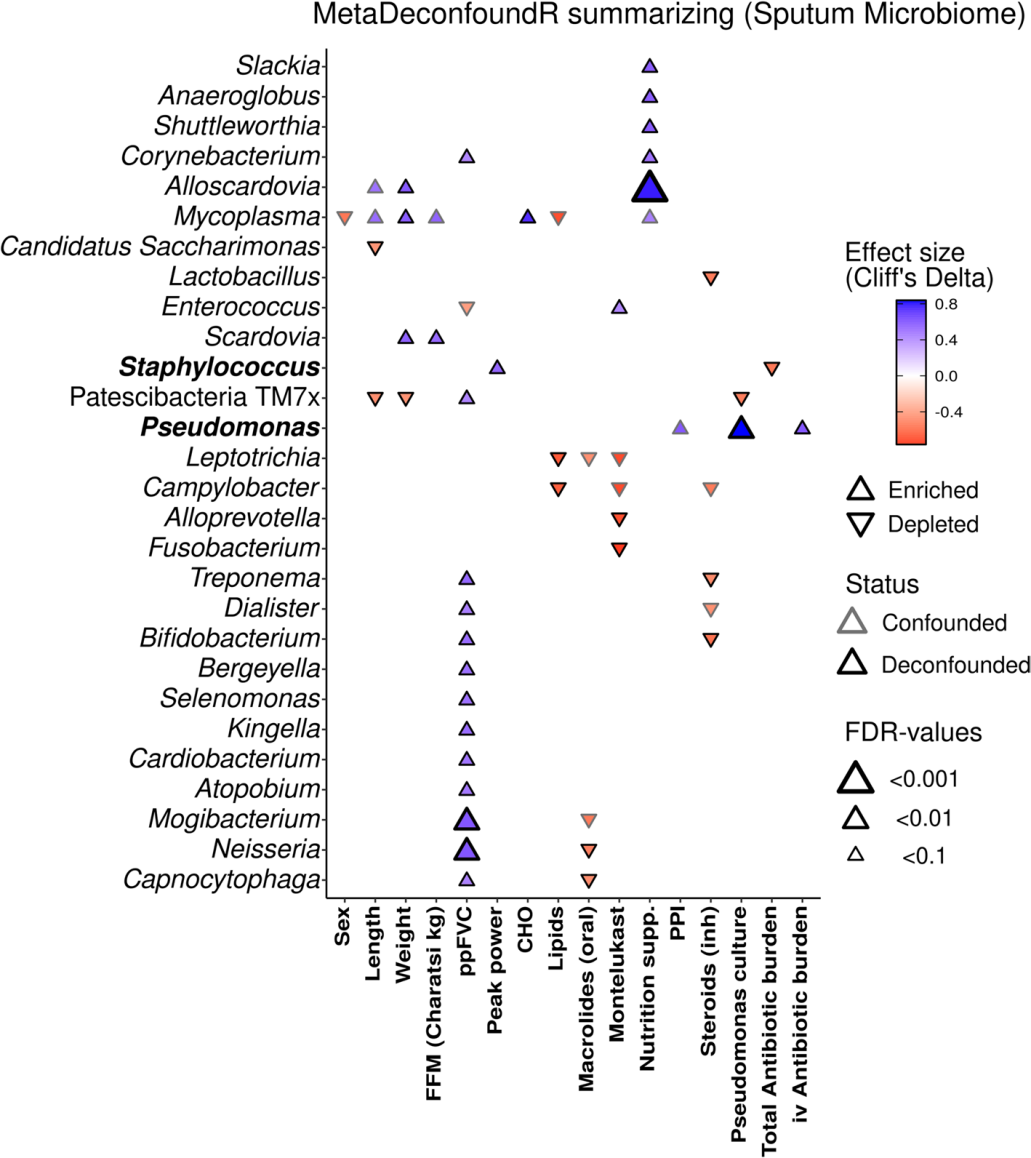
Association of microbial features to clinical and nutritional covariates from sputum microbiome. The plot shows bacterial taxa, at genus levels, altered significantly (post hoc FDR < 0.05) in abundance and showing a study effect, compared to baseline and follow-up visits. Signed effect sizes are shown through marker color. Direction of cuneiform plot indicates if taxa were enriched or depleted within the covariate groups (e.g. pwCF with oral Macrolide treatment displayed lower abundance of Neisseria, higher ppFVC correlated positively with increased *Neisseria* etc.). Size of cuneiform plots indicate significance. Covariates not shown had no difference in rank-transformed values (Cliff’s delta = 0). PPI = proton pump inhibitors, inh = inhalative, iv = intravenous

We analyzed the complete dataset to identify associations between clinical data and the abundance of bacterial taxa in the specific microbiomes.

In sputum, a higher abundance of *Neisseria* and *Mogibacterium* and other low abundant taxa correlated with higher ppFVC but not ppFEV1(Fig. 2). CFTR-modulator Lumacaftor/Ivacaftor therapy (N = 6) had no relevant effect on sputum bacteria. Inhaled antibiotics (*N* = 10) did not impact genera abundance in sputum. However, oral macrolides (*N* = 4) were associated with lower Neisseria abundance*. Staphylococcus* abundance in sputum was negatively associated with total antibiotic burden. IV-antibiotic burden correlated positively with *Pseudomonas* abundance in sputum.

In the stool microbiome (Supplementary Fig. 7), *Lachnospiraceae UCG:004* showed a significant positive association with higher FFM measurements. We observed that regular intake of the stool softener Polyethylene Glycol (*N* = 4) was associated with an increased abundance of several genera, like *Olsenella* or *Collinsella*. Antibiotic burden did not correlate with specific genera abundance (Supplementary Fig. 7). Among the nutritional characteristics from the cohort, higher fiber intake correlated positively with *Solobacterium* abundance in the stool. *Lactobacillus* abundance did not increase during probiotic supplementation (Supplementary Fig. 8).

### Recent antibiotic treatment, but not long-term cumulative antibiotic burden, affects diversity and richness

**Fig. 3:**
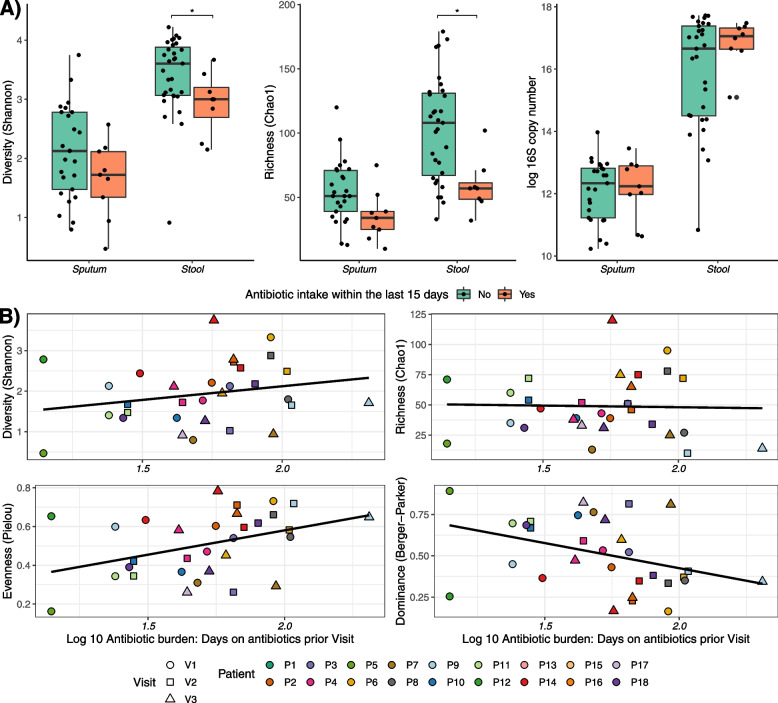
**A** Impact of antibiotic intake within the last 15 days prior sampling on alpha diversity measures and 16S copy number. Asterisks indicate significance for LMMs with Benjamini–Hochberg correction for multiple testing (FDR: ‘*’ < 0.05). **B** Relation between antibiotic burden and alpha diversity measures in sputum. Antibiotic burden had a significant impact on sputum evenness

We used generalized linear mixed models (GLMM), adjusting for repeated measures by subject (Patient_number) and visit as random factors, to assess how antibiotic intake impacts the stool and sputum microbial composition. Shannon diversity and genus richness were significantly reduced in stool samples from patients receiving antibiotics in the last 15 days prior to sampling (LRT: χ^2^ = 6.79 (diversity) and 7.24 (richness), FDR < 0.05*) (Fig. 3A, Supplementary Table 6). In sputum samples this effect did not reach the significance threshold. Bacterial load (in 16S sRNA copy number) of sputum and stool was not reduced by oral or IV-antibiotic treatment taken within the last 2 weeks prior to sampling (Fig. 3A).

Despite not reaching significance, we observed that changes in total antibiotic burden might explain around 10% of the microbial composition changes among stool samples (LRT: χ^2^ = 1.46, *p* = 0.226), and in contrast, just around 1% among sputum samples (LRT: χ^2^ = 0.1674, *p* = 0.682). Antibiotic burden did not affect alpha diversity measures in stool samples. In sputum, evenness increased with higher antibiotic burden (LRT: χ^2^ = 9.907, fFDR < 0.05*)(Fig. 3B, Supplementary Table 7). IV-antibiotics alone had no significant impact on diversity measurements, neither in stool nor in sputum.

## Discussion

We hypothesized that exercise and nutritional intervention would improve health in pwCF, which could be traced in their microbiomes. So far, microbiome modulation via exercise therapy has not been studied in CF. This study demonstrates the high resilience of the CF respiratory and intestinal microbiota to supportive and antibiotic treatment.

The resilience of the microbiome, defined by the ability to remain or return to a stable state [25], was demonstrated by the lack of significant shifts in intra- or inter-individual microbial composition during our intervention at three sample time points. In a healthy host-microbiome ecosystem, resilience is considered to be beneficial to maintain equilibrium during external perturbations and can confer protection from disease [25]. We demonstrate that the resilience of the CF respiratory microbiome is driven by the high stability and dominance of disease-associated pathogens.

Participants successfully increased weekly training duration and frequency by about 40% due to the offered program [22], which underlines the efficacy of the study. Considering that VO2peak remained stable while lung function declined (as expected in pwCF), highlights further benefit of the sports intervention for the participants. Despite this significant lifestyle change, the respiratory and intestinal microbiomes remained highly resistant to the intensified exercise. This is in contrast to previous studies in prediabetic, diabetic or obese individuals [26, 27], which linked sport to a healthier gut microbiome composition. In those studies, effective sports intervention substantially improved cardiorespiratory fitness or body composition. Our cohort increased fat-free mass by intensified training, but not cardiorespiratory fitness (VO2peak) or lung function. An increase in FFM serves as a proxy for muscle gain [28] and demonstrates the additional efficacy of the offered program. Hence as muscle and fat tissues are less affected by the disease genotype, modulation of those is more feasible for pwCF, whereas VO2peak and lung function are strongly linked to CF lung disease and are less susceptible to exercise. In healthy populations, intensified exercise did not influence gut microbiota. Moitinho-Silva et al. [29] and Wang et al. [30] offered similar training, endurance and strength exercise programs to healthy adult and adolescent participants. Their intervention periods were much shorter (6 or 12 weeks, respectively), but they also found no changes in the gut microbiota related to exercise. One might hypothesize that prolonged intensified exercise and relevant changes in cardiorespiratory fitness are needed to influence the gut microbiota. In contrast, summarizing results of acute exercise interventions, Clauss et al. [20] concluded that intestinal bacterial diversity increased with the intensity of the physical activity performed. This included higher abundance of short-chain fatty acids producing bacteria, which might confer anti-inflammatory benefits to the host. The intensity of the physical activity performed by our study participants might not have been sufficient to modulate microbial composition, due to the underlying disease. The authors also noted a lack of longitudinal studies, especially considering nutritional intake and host characteristics as major confounders. Within our longitudinal study, we could control for confounding effects of nutritional variation, medication and subjects’ characteristics as BMI, FFM and disease severity.

No studies on the impact of exercise on the respiratory microbiome have been published so far. There is well established evidence that disease-associated pathogens are dominating the CF respiratory microbiome [11, 24]. Despite this distinct clustering of dominant taxa, changes and abundance of other genera within the sputum remained highly individual (Fig. 1E). Frey et al. [24] showed similar clustering of CF sputum into *Pseudomonas*, *Staphylococcus,* and an oropharyngeal-like flora (OF), including *Streptococcus-* and *Neisseria-* dominated communities. As in our cohort, *Pseudomonas-* and *Staphylococcus*-dominated microbiomes displayed lower Shannon diversity when compared to the OF microbiome. The OF microbiome can be compared to our cohort’s *Streptococcus* dominated microbiomes, which displayed higher diversity and species richness (Fig. 1C, D, E), thus implying a healthier respiratory microbiome in those individuals. Similar dominance patterns have also been found in sinus secretion of pwCF with chronic rhinosinusitis [31]. In both studies [24, 31], as in our study, individuals with a *Pseudomonas*-dominated respiratory microbiome had the poorest lung function (ppFEV_1_). The overgrowth of pathogens has been linked with disease severity several times [11, 24, 32]. Antibiotic treatment to reduce or eradicate those pathogens from the lungs is the standard of care in CF.

We showed how antibiotic intake affects the microbiome both in sputum and stool only transiently. We found that recent antibiotic treatments decreased the diversity and richness of the intestinal microbiome, and to some extent, of the respiratory microbiome (Fig. 3A).

Interestingly, bacterial biomass (measured in 16S copies) did not change due to antibiotic intake. Hence, the abundance of dominant pathogenic taxa was little affected by antibiotic intake. A reduction of less-abundant “satellite taxa” might explain the decrease of diversity and richness. Similar results were shown by Cuthbertson et al. [33] and Fodor et al. [34], who both analyzed the CF sputum microbial composition during exacerbation and after antibiotic treatment. Nelson et al. showed that inhaled tobramycin affected low abundant genera but not *Pseudomonas* abundance [35], thus supporting our results. *Pseudomonas* abundance was stable in our cohort in individuals with continuous inhaled antibiotic treatment.

The observed resilience of the respiratory microbiome to antibiotic treatment is in line with previous studies. Bacci et al. [36] showed in a 15 months-longitudinal study, how the pathogenic microbiome signature of the lung is recovered after antibiotic treatment, demonstrating the ability of the CF respiratory microbiome to return to stable composition after perturbation. In cardiometabolic diseased and healthy adults, a higher number of antibiotic courses within the last 5 years correlated with lower intestinal microbiota diversity [37]. We could not reproduce this observation in pwCF. Long-term antibiotic burden did not affect alpha diversity measures and further emphasizes the resilience of CF microbiomes (Fig. 3B and Supplementary Table 7). This might be explained by an already established dysbiotic composition at study start in the respiratory and intestinal microbiome of our cohort. Thus, we might not detect subtle changes due to antibiotic intake in our cohort. Burke et al. showed that number of iv-antibiotic courses in the year prior sampling can affect intestinal microbiome alpha-diversity and taxonomic abundances in their cohort of pwCF [38]. In addition, they pointed out, how the intense medical treatment of pwCF can promote intestinal dysbiosis. The dysbiotic state of the CF intestinal microbiome has been recently highlighted again [39]. We attempted to modulate intestinal microbiome composition via a probiotic intervention. Intriguingly, despite *Lactobacillus* intake, the abundance of it remained stable. Previous studies reported that lactic acid bacteria might fail to colonize the human gut depending on the hosts’ baseline gut microbiome characteristics [40]. Further studies are needed to investigate if only certain pwCF might benefit from probiotic supplementation, depending on baseline microbiome structures.

As part of the limitations of the current study, the limited number of pwCF that finalized the program is a major. In view of the expected high degree of compositional variability, we might have failed to detect more subtle signals due to limited sample numbers. Despite the limited cohort size, the repeated sampling allowed us to test our hypothesis with confidence. Even though the taxonomic resolution of 16S rRNA gene amplicon sequencing is limited, we showed that dominant CF-pathogens can be detected at the genus level. In the future, larger cohort studies, deploying whole genome sequencing, should overcome these limitations to study the putative effects of exercise and nutrition on the intestinal and respiratory microbiota in pwCF.

## Conclusions

In summary, there is a lack of effective microbiome-modulating therapies to overcome the vigorous pathogen-dominated signature of the CF respiratory microbiome. The recently approved highly effective CFTR-modulator therapeutics might have the potential to surpass the demonstrated resilience of CF microbiomes. Future studies on CF microbiome composition under these therapeutics are needed.

## Methods

### Study design

An online monitored personalized exercise and nutrition program was offered to pwCF. Participants were enrolled between August 2016 and February 2019 at the University Medical Center in Mainz, Germany. Inclusion criteria were age > 12 years at study start and forced expiratory volume in one sec FEV_1_% predicted-values (ppFEV_1_) > 28 and < 100 and/or lung clearance index (LCI) > 9. Patients were excluded if they met orthopedic, cardiovascular or neurological contraindications regarding the sports program. Study start was delayed if a patient suffered severe pulmonary exacerbation or acute infection.

### Interventions

The exercise program combined strength and endurance training and was performed in the home-environment of the patients. Participants reported their training progress and received weekly adjusted training recommendations by a sports scientist via an online platform. Details regarding the exercise intervention were recently published by Hillen et al. [22].

For the 3- day weighed food record (3d-WFR), participants weighed their dietary intake for three consecutive days prior V2 and V3. The daily intake of calories and macronutrients were quantified via an electronic dietary assessment tool (PRODI). Based on the results from the 3d-WFR, patients received nutrition counseling from a dietician to improve their diet according to CF-guidelines. Every patient took probiotics (*Lactobacillus rhamnosus* LGG Infectopharm®) from V2 to V3. When probiotics were part of the patient’s diet, they discontinued their use four weeks before the study started.

### Clinical assessments

To monitor participants during the study, 3 clinical visits took place. Visit 1 (V1) at baseline, visit 2 (V2) after 3 months and visit 3 (V3) after 12 months (at the end of the study).

At each visit *lung function* (ppFEV_1_ and ppFVC (forced vital capacity in percent-predicted-values)) was assessed via flow- volume spirometry (MasterScope®Body, CareFusion, Germany) in line with the criteria of the American Thorax Society/European Respiratory Society. *Cardiopulmonary exercise testing* was performed on a treadmill with stepwise increment of speed (km/h) and incline (%) until maximum exhaustion was reached to assess peak oxygen uptake (VO2_max_)_._ Further details are published by B. Hillen et al. [22].

To better evaluate body mass composition, *bioelectrical impedance analysis* (BIA) was performed to assess fat free mass. At each visit 3 measurements were taken, means were calculated and fat free mass in kg estimated using the CF-specific equation published by Charatsi et al. [41].

*Disease severity* was defined as mild (ppFEV_1_ > 70) and moderate (ppFEV_1_ 40–70), choosing the highest ppFEV_1_ for each participant within three months prior to the study start. *Antibiotic intake* was retrospectively assessed for the 12 months prior to the study start and during the study. The number of treatment days and administration routes were collected. Antibiotic burden prior visit was defined by days on antibiotics during the last 365 days before sampling (e.g. 14 days on ceftazidime + colistin = 14 days).

### Microbiome sampling, DNA-extraction and 16S sRNA gene sequencing, absolute quantification of bacterial load by qPCR

The intestinal and respiratory microbiome was characterized at each visit (V1-V3). Respiratory microbiome was evaluated from sputum; for intestinal microbiome stool microbiome was inferred as a proxy. Stool sampling was done at home with a provided stool sampling kit, as described in Knoll/Forslund et al. [42]. Collected stool samples were stored at home between 4 °C and 8 °C and transferred to the clinic within 24 h. Spontaneously expectorated sputum was collected into a sterile container during each visit at our CF-department. In the CF-department, both sample types were frozen at -80 °C immediately until further processing. For both sample types no preservation media were used. The number of participant samples analyzed varied depending on sample availability.

DNA was extracted and the V4 region of the 16 s rRNA genes was amplified using the primers 515F and 806R. For all samples, 16S copies/ml of DNA were estimated by qPCR.

For detailed DNA extraction and sequencing methods please refer to the supplement. Sequencing data is available at NCBI BioProject PRJNA810321.

### Bioinformatic analysis

Sequences were pre-processed to infer amplicon sequence variants (ASVs) following the DADA2 v1.18 pipeline using R v. 4.03. All the R scripts used for this analysis are available at https://github.com/VictorHJD/exercise-cf-intervention.git. Details on sequence processing and statistical analyses are in the supplementary methods. All analyses were controlled for false discovery rate (FDR), employing the Benjamini–Hochberg procedure.

To assess alpha diversity metrics, we used Chao1, Shannon, Pielou and Berger-Parker indices to estimate richness, diversity, evenness and dominance. Beta diversity was assessed using the Bray–Curtis dissimilarity index between samples. We compared alpha diversity metrics and beta diversity distances between visits and dominant taxa for each sample type via Mann–Whitney U tests. We used Principal Coordinates Analysis (PCoA) for multivariate analysis with the Bray–Curtis distances. Permutational analysis of variance (PERMANOVA) for multivariate effect was performed, stratified for patient ID. We identified the dominant taxa from each patient as the ASV with highest relative abundance of all ASVs at the genus level per sample. Functional profiles were predicted from the 16S rRNA sequences using PICRUSt2 [43].

Generalized linear mixed-effects models (GLMM) were calculated with patient ID and visit (where appropriate) as random factors for different inference analyses. Pairwise comparisons for the interactions were calculated. GLMMs were compared through a likelihood ratio test (LRT).

To assess possible associations between ASVs abundance and clinical characteristics, nutritional information, or medication, we ran a multivariate correlation analysis [44]. In brief, we tested for correlations for each microbial feature with each covariate and used Spearman’s rho as a standardized signed effect estimate. A post-hoc test was done to account for dependency between patient samples: for each of two correlated features, a mixed-effects model was fitted of the rank-transformed variable using the rank of the other as an explanatory variable, with patient ID as a random effect. We reported standardized nonparametric effect sizes (signed) Cliff’s delta metric.

Changes on clinical parameters were analyzed with repeated measures ANOVA.

## Supplementary Information

**Additional file 1.**

## Data Availability

The datasets supporting the conclusions of this article are available in NCBI BioProject PRJNA810321 https://www.ncbi.nlm.nih.gov/bioproject/PRJNA810321.

## Abbreviations

CF: Cystic fibrosis
CFTR: Cystic fibrosis transmembrane conductance regulator
pwCF: People with cystic fibrosis
ppFEV1: Forced expiratory volume in first second in percent-predicted-values
ppFVC: Forced vital capacity in percent-predicted-values
LCI: Lung clearance index
BMI: Body mass index
IV: Intravenous
ASV: Amplicon sequence variant
SD: Standard deviation
ANOVA: Analysis of variance
IQR: Inter quartile range
qPCR: Quantitative polymerase chain reaction
PCoA: Principal coordinates analysis
FDR: False discovery rate
GLMM: Generalized linear mixed-effects models
LRT: Likelihood ratio test
