# Supplementary material for: Resilience and stability of the CF- intestinal and respiratory microbiome during nutritional and exercise intervention
Source: BMC Microbiol. 2023 Feb 21;23:44. doi: 10.1186/s12866-023-02788-y (PMC9942320; doi:10.1186/s12866-023-02788-y)
Supplement: Supplementary file 1 — Additional file 1. [file 12866_2023_2788_MOESM1_ESM.docx]

1. **Supplementary Figures and Tables:**


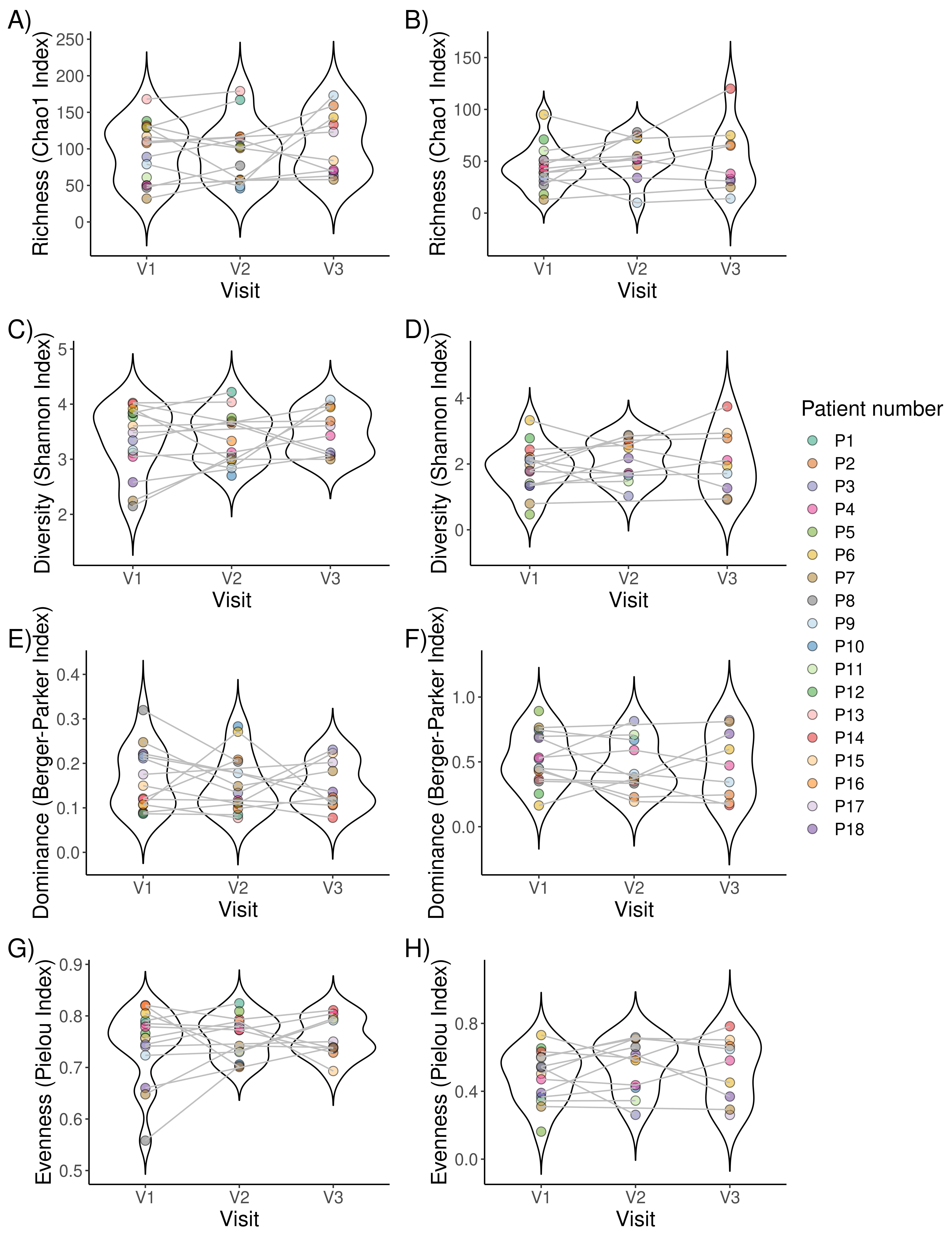
**Supplementary Figure 1.** The intervention had no significant (two-sided MWU *P* > 0.05) impact neither on stool (A, C, E, G) nor sputum (B, D, F, H) microbiome alpha diversity (Chao1 richness, Shannon diversity, Berger-Parker Dominance and Pielou’s Evenness) across observation times V1–V3. Each color represents samples from the same patient and gray lines connect samples from the same patient.


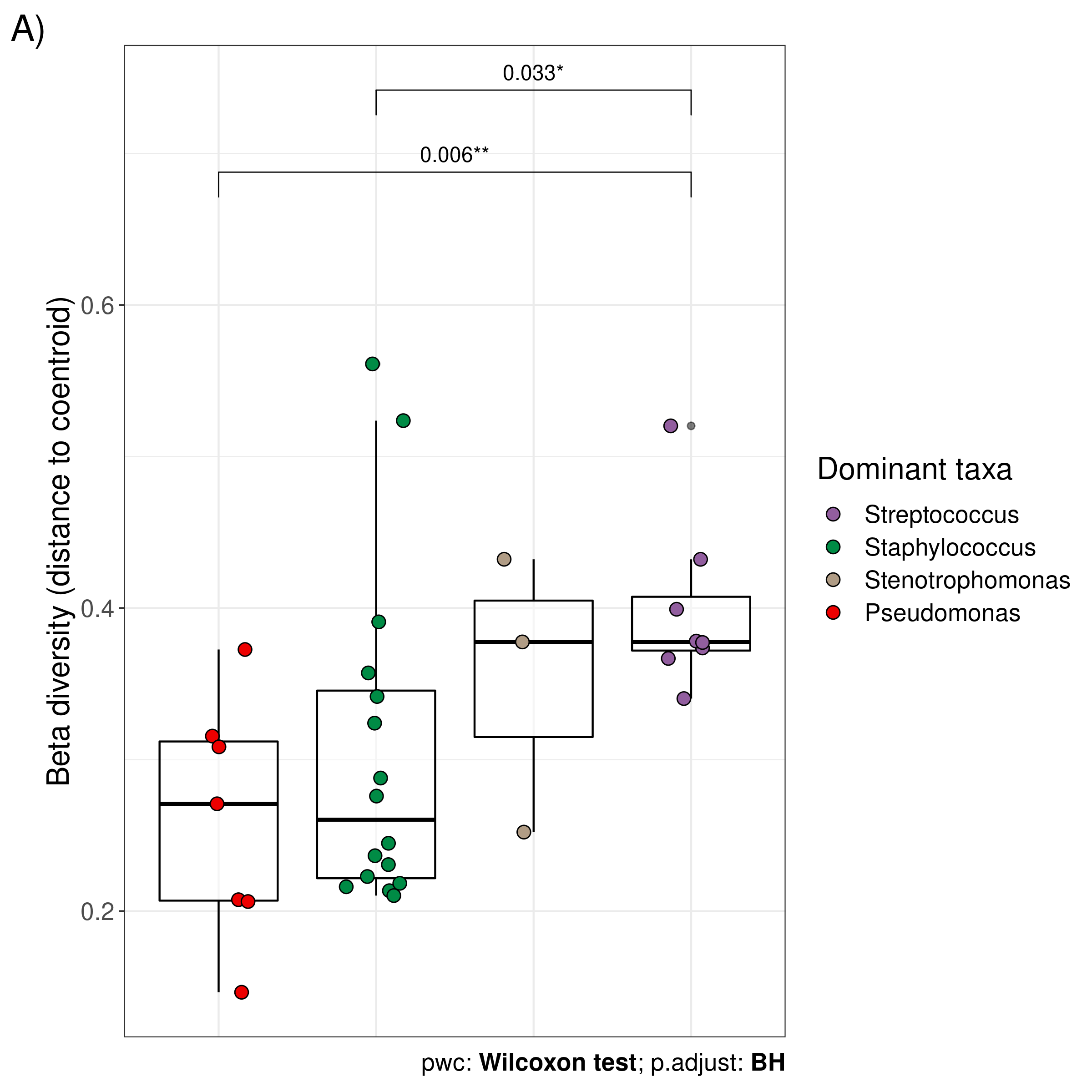
**Supplementary Figure 2.** Intra-cluster sample variability represented by the distance to each cluster centroid. Distances between dominant taxa cluster were compared by Mann–Whitney U tests with Benjamini-Hochberg correction for multiple testing (Signif. codes:  0 ‘***’ 0.001 ‘**’ 0.01 ‘*’ 0.05 ‘.’ 0.1).


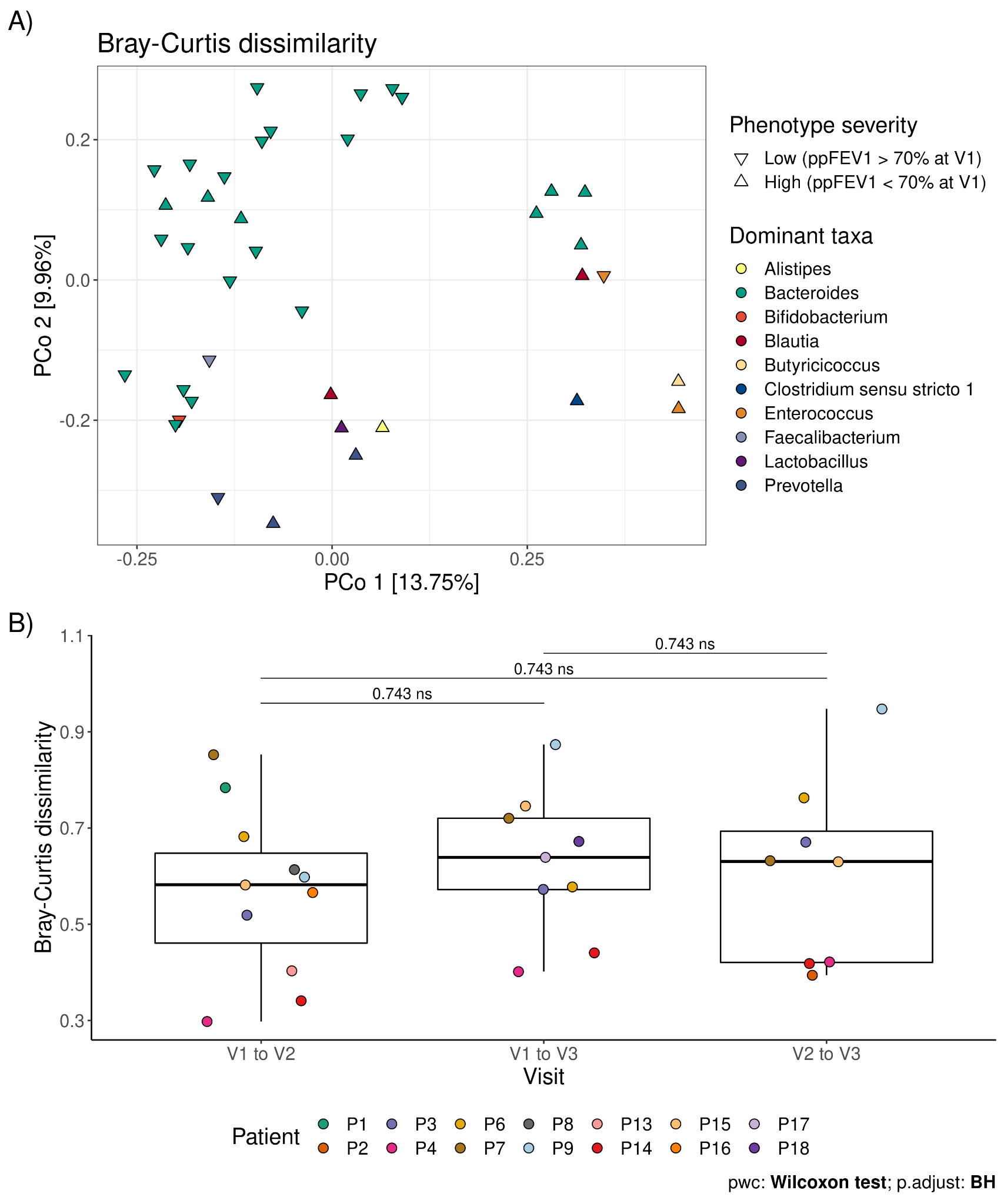


**Supplementary Figure 3.** Differences in composition in stool microbiome were independent from the intervention or severity of the disease between patients (A) or within patients between visits (B). In panel A, points are colored by the dominant taxa detected and the shape by the severity classification based on ppFEV1 at visit 1. For panel B, each color represents samples from the same patient.

**
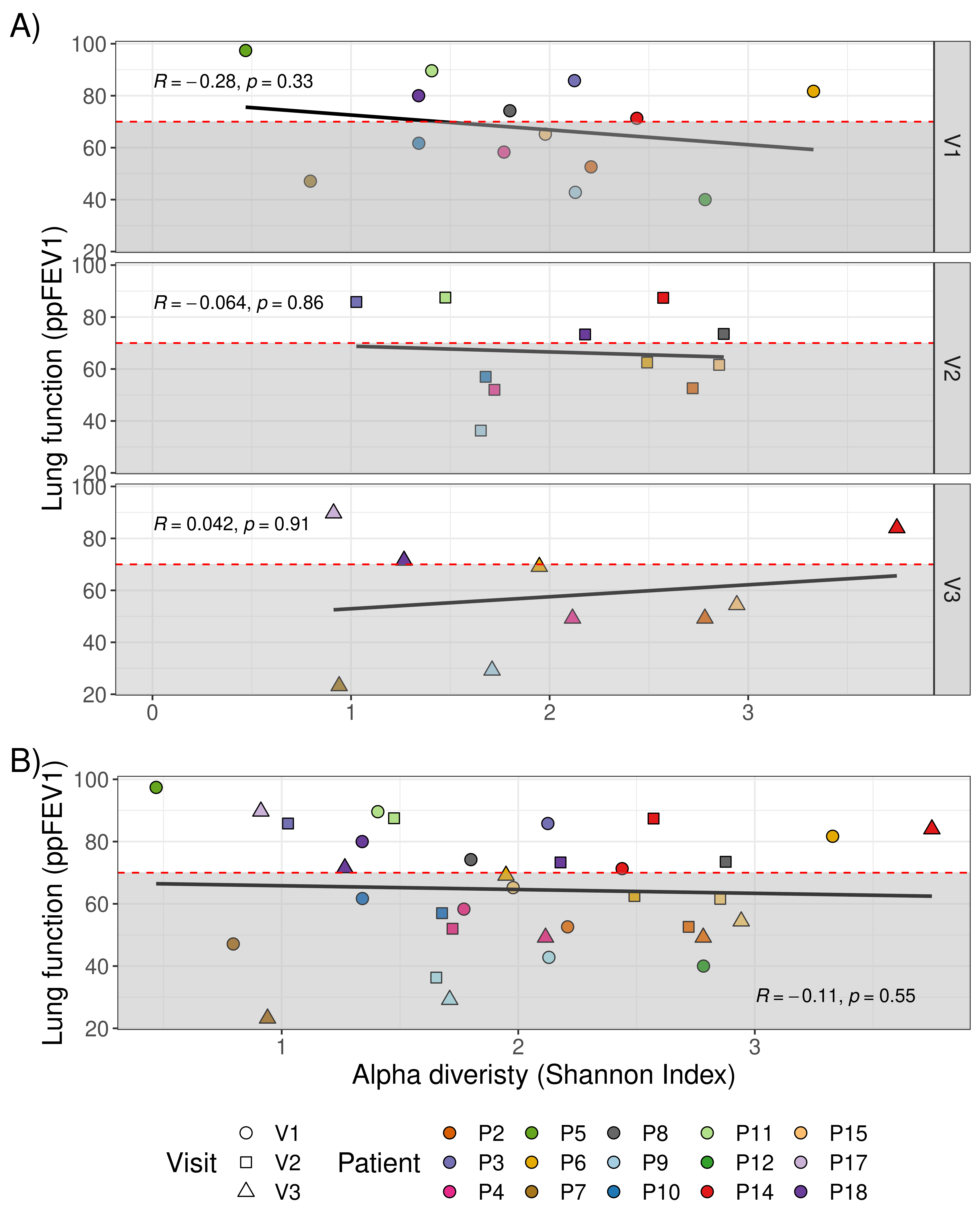
**

**Supplementary Figure 4.** Impact of sputum microbiome alpha diversity on lung function. A) between visits. B) cumulative. The dashed red line indicates ppFEV_1_ of 70, dividing into mild and moderate disease severity, respectively.


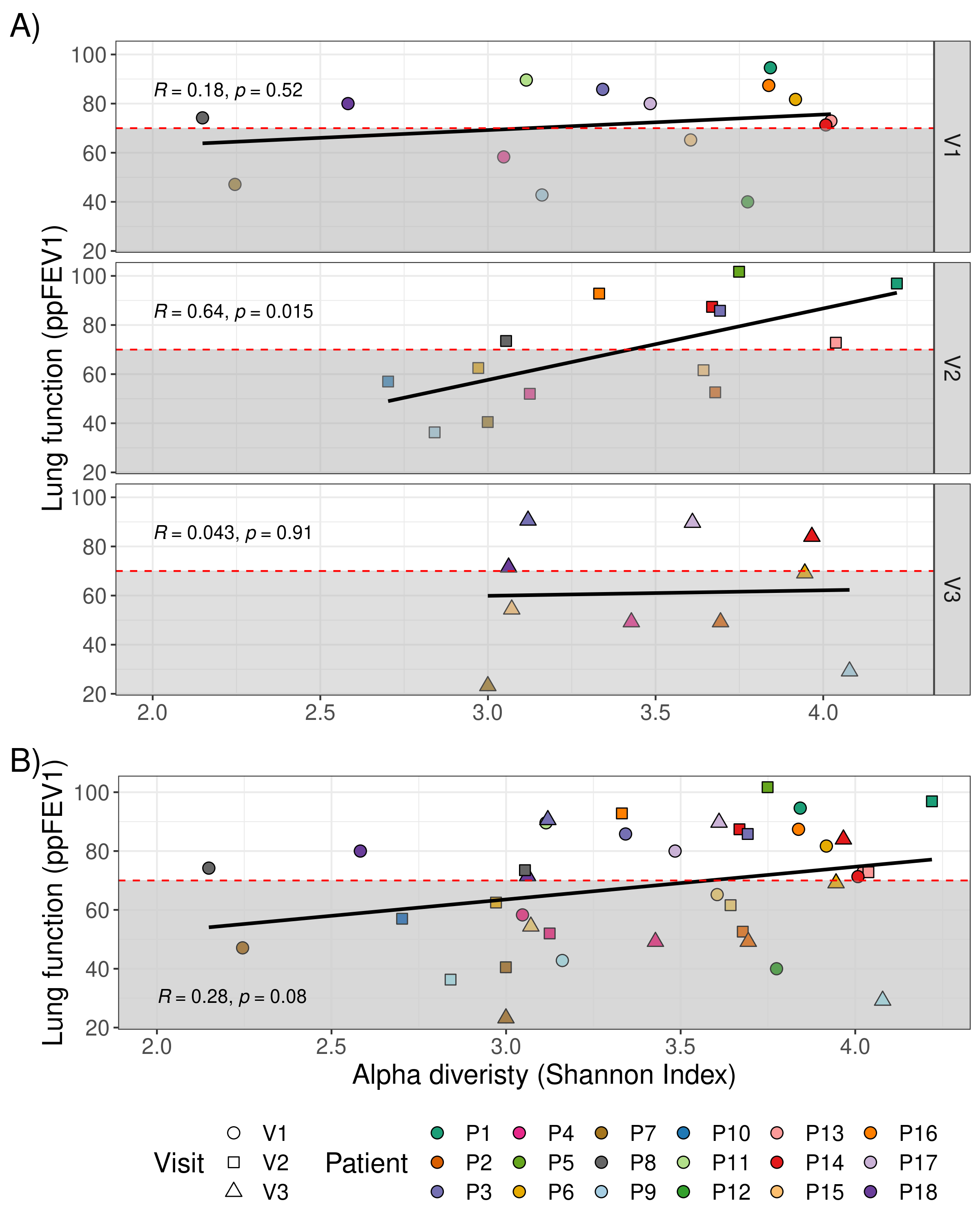


**Supplementary Figure 5.** Impact of stool microbiome alpha diversity on lung function. A) between visits. B) cumulative.


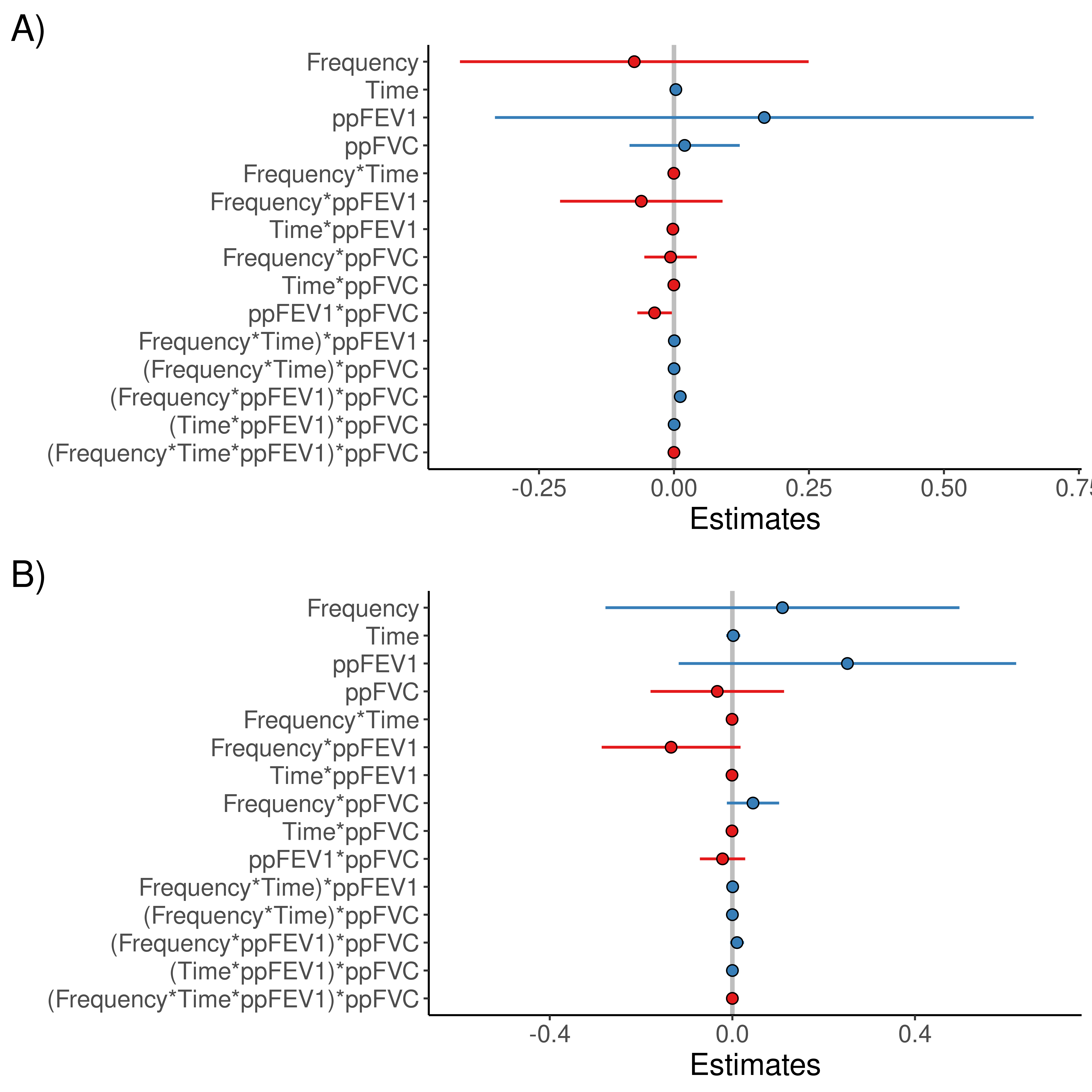


**Supplementary Figure 6.** Generalized linear mixed model to predict the effect of training frequency and time, lung function parameters and their interaction on microbial composition in stool/sputum of cystic fibrosis patients, A) Stool and B) Sputum. We performed GLMMs with the with Bray-Curtis distances as our proxy of microbial composition as response variable and training frequency and time and ppFEV1 and ppFVC as lung function measurements. Both models had patient id as random effect to control for pseudo-replication. Each panel contains the estimate for each fixed effect or interaction (dots) and its standard error (bars). Blue dots and bars represent positive estimates, while those colored in red, negative estimates.

**Supplementary Figure 7.** Association of clinical metadata and abundance of genera in the stool microbiome.


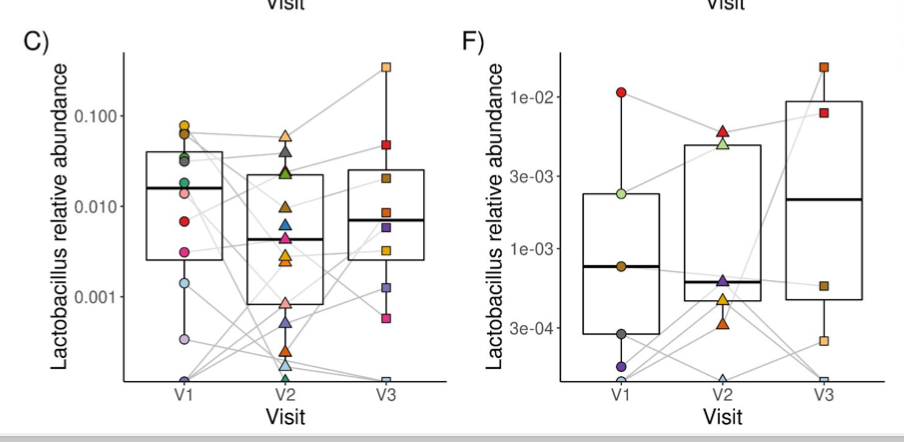


**Supplementary Figure 8.** Relative abundance of *Lactobacillus* in stool (C) and sputum (F) microbiomes between visits.

**Supplementary Table 1: Macronutrient intake within the study cohort**

| **Macronutrient intake** | **Visit 2** | **Visit 3** |
| --- | --- | --- |
| **n =** | 16 | 13 |
| **Calories (kcal/kg/d)** |  |  |
| **Median ± SD** | 42,67±13,37 | 42,9±13,14 |
| **Range** | 27,2-68,6 | 18,8-63,1 |
| **Carbohydrates (in %)** |  |  |
| **Median** | 41,5 | 43 |
| **Interquartile range** | 37,5-46,5 | 36,8-52,2 |
| **Range** | 33-59 | 33-62 |
| **Fat (in %)** |  |  |
| **Median** | 37,5 | 38 |
| **Interquartile range** | 32,4-41,8 | 29,5-45,5 |
| **Range** | 25-46 | 16-50 |
| **Proteins (in %)** |  |  |
| **Median** | 16,2 | 14,7 |
| **Interquartile range** | 14,3-20,9 | 13,7-17,8 |
| **Range** | 13-25 | 12-33 |
| **Fibre (in %)** |  |  |
| **Median** | 1,5 | 1,33 |
| **Interquartile range** | 1-2 | 1-1,7 |
| **Range** | 1-3,3 | 1-2,3 |
| **Alcohol (in %)** |  |  |
| **Median** | 0 | 0 |
| **Interquartile range** | 0-1,25 | 0-1,17 |
| **Range** | 0-5 | 0-4 |

**Supplementary Table 2. Effect of training frequency and time and lung function parameters to infer microbial composition in stool of cystic fibrosis patients.**

| GLM: Bray-Curtis dissimilarity~ Training frequency * Training time * ppFEV1 * ppFVC + (1\|Patient_number) | | | | | |
| --- | --- | --- | --- | --- | --- |
| Coefficients | Estimate | Std error | t value | Pr(>\|t\|) | fdr |
| Intercept | 0.4440 | 0.4353 | 1.020 | 0.347 | 0.6456 |
| (Training) Frequency | -0.0735 | 0.1320 | -0.557 | 0.598 | 0.6830 |
| (Training) Time | 0.0035 | 0.0035 | 1.000 | 0.356 | 0.6456 |
| ppFEV1 | 0.1673 | 0.2039 | 0.820 | 0.443 | 0.6468 |
| ppFVC | 0.0197 | 0.0417 | 0.473 | 0.653 | 0.6966 |
| Frequency * Time | -0.0003 | 0.0004 | -0.718 | 0.465 | 0.6468 |
| Frequency * ppFEV1 | -0.0605 | 0.0615 | -0.984 | 0.363 | 0.6456 |
| Time * ppFEV1 | -0.0020 | 0.0012 | -1.714 | 0.137 | 0.3663 |
| Frequency * ppFVC | -0.0063 | 0.0199 | -0.319 | 0.761 | 0.7609 |
| Time * ppFVC | -0.0002 | 0.0005 | -0.597 | 0.572 | 0.6830 |
| ppFEV1 * ppFVC | -0.0360 | 0.0131 | -2.748 | 0.033* | 0.3489 |
| Frequency * Time * ppFEV1 | 0.0006 | 0.0003 | 1.881 | 0.109 | 0.3489 |
| Frequency * Time * ppFVC | 0.0001 | 0.0002 | 0.744 | 0.485 | 0.3489 |
| Frequency * ppFEV1 * ppFVC | 0.0116 | 0.0048 | 2.406 | 0.053 | 0.3489 |
| Time * ppFEV1 * ppFVC | 0.0002 | 0.0001 | 2.014 | 0.091 | 0.3489 |
| Frequency * Time * ppFEV1 * ppFVC | -0.00008 | <0.0001 | -2.176 | 0.073 | 0.3489 |

---

Signif. codes: 0 ‘***’ 0.001 ‘**’ 0.01 ‘*’ 0.05 ‘.’ 0.1 ‘ ’ 1

**Supplementary Table 3. Effect of training frequency and time and lung function parameters to infer microbial composition in sputum of cystic fibrosis patients.**

| GLM: Bray-Curtis dissimilarity~ Training frequency * Training time * ppFEV1 * ppFVC + (1\|Patient_number) | | | | | |
| --- | --- | --- | --- | --- | --- |
| Coefficients | Estimate | Std. Error | t value | Pr(>\|t\|) | fdr |
| Intercept | 0.01049 | 0.31789 | 0.03301 | 0.97459 | 0.97459 |
| (Training) Frequency | 0.10973 | 0.16397 | 0.66918 | 0.52483 | 0.86318 |
| (Training) Time | 0.00193 | 0.00655 | 0.29396 | 0.77732 | 0.88836 |
| ppFEV1 | 0.25204 | 0.15632 | 1.61229 | 0.15093 | 0.63383 |
| ppFVC | -0.03301 | 0.06186 | -0.53362 | 0.61012 | 0.86318 |
| Frequency*Time | -0.00064 | 0.00171 | -0.37490 | 0.71884 | 0.88472 |
| Frequency*ppFEV1 | -0.13418 | 0.06433 | -2.08594 | 0.07542 | 0.63383 |
| Time*ppFEV1 | -0.00080 | 0.00150 | -0.53587 | 0.60865 | 0.86318 |
| Frequency*ppFVC | 0.04519 | 0.02422 | 1.86576 | 0.10432 | 0.63383 |
| Time*ppFVC | -0.00085 | 0.00087 | -0.97427 | 0.36238 | 0.81386 |
| ppFEV1*ppFVC | -0.02150 | 0.02101 | -1.02335 | 0.34020 | 0.81386 |
| Frequency*Time*ppFEV1 | 0.00058 | 0.00044 | 1.33435 | 0.22386 | 0.71635 |
| Frequency*Time*ppFVC | 0.00004 | 0.00026 | 0.16509 | 0.87354 | 0.93178 |
| Frequency*ppFEV1*ppFVC | 0.01023 | 0.00648 | 1.57850 | 0.15846 | 0.63383 |
| Time*ppFEV1*ppFVC | 0.00009 | 0.00019 | 0.47775 | 0.64738 | 0.86318 |
| Frequency*Time*ppFEV1*ppFVC | -0.00005 | 0.00005 | -0.88222 | 0.40693 | 0.81386 |

**Supplementary Table 4. Linear model impact of alpha diversity in lung function (stool)**

| **Response (ppFEV1)** | **Sum sq.** | **Df** | **F value** | **Pr(>F)** |
| --- | --- | --- | --- | --- |
| *Intercept* | 1178.4 | 1 | 3.113 | 0.0869 |
| *Diversity* (Shannon Index) | 216.0 | 1 | 0.571 | 0.455 |
| *Visit* | 1106.9 | 2 | 1.462 | 0.246 |
| *Interaction Diversity-Visit* | 1149.8 | 2 | 1.519 | 0.234 |
| Residuals | 12491.8 | 33 |  |  |

**Supplementary Table 5. Linear model impact of alpha diversity in lung function (sputum)**

| **Response (ppFEV1)** | **Sum sq.** | **Df** | **F value** | **Pr(>F)** |
| --- | --- | --- | --- | --- |
| *Intercept* | 11524.6 | 1 | 29.809 | <0.001 |
| *Diversity* (Shannon Index) | 242.8 | 1 | 0.628 | 0.435 |
| *Visit* | 772.7 | 2 | 0.999 | 0.381 |
| *Interaction Diversity-Visit* | 406.9 | 2 | 0.526 | 0.597 |
| Residuals | 10825.1 | 28 |  |  |

**Supplementary Table 6. Impact of recent antibiotic treatment on alpha diversity measurements on sputum and stool microbiomes.**

| ***Full Model 1:***  Dependent variable ~ antibiotics last 15d + ppFEV1 + (1\|Patient_number) + (1\|Visit) | | | | |
| --- | --- | --- | --- | --- |
| ***Null Model 1:***  Dependent variable ~ ppFEV1 + (1\|Patient_number) + (1\|Visit) | | | | |
| **Dependent variable** | **Intercept**  antibiotics last 15d | **LRT: χ^²^** anova(full, null) | **p**  anova(full, null) | **fdr** |
| **Sputum** | | | | |
| Shannon diversity | -0.282138 | 1.4132 | 0.2345 | 0.5862500 |
| Genus richness (observed) | -14.8430 | 4.1599 | **0.04139** | 0.2069500 |
| Evenness (Simpson) | 0.019662 | 0.533 | 0.4654 | 0.7756667 |
| Dominance (Berger-Parker) | 0.34371 | 0.1437 | 0.7046 | 0.8807500 |
| 16 S copy number | 44010 | 1e-04 | 0.99 | 0.9900000 |
| **Stool** | | | | |
| Shannon diversity | -0.514659 | 6.7854 | **0.009191** | **0.0229775** |
| Genus richness (observed) | -42.068406 | 7.2413 | **0.007125** | **0.0229775** |
| Evenness (Simpson) | 1.149e-02 | 0.4561 | 0.4995 | 0.5953000 |
| Dominance (Berger-Parker) | 0.0291820 | 1.1645 | 0.2805 | 0.4675000 |
| 16 S copy number | 2937633 | 0.2821 | 0.5953 | 0.5953000 |

**Supplementary Table 7. Impact of long-term antibiotic burden on alpha diversity measurements on sputum and stool microbiomes.**

| ***Full Model 1:***  Dependent variable ~ Antibiotic Burden_total+ ppFEV1 + (1\|Patient_number) + (1\|Visit) | | | | |
| --- | --- | --- | --- | --- |
| ***Null Model 1:***  Dependent variable ~ ppFEV1 + (1\|Patient_number) + (1\|Visit) | | | | |
| **Dependent variable** | **Intercept/Estimate**  Antibiotic Burden_total | **LRT: χ^²^** anova(full, null) | **p**  anova(full, null) | **fdr** |
| **Sputum** | | | | |
| Shannon diversity | 0.001630 | 0.246 | 0.620 | 0.827 |
| Genus richness (observed) | - 0.03865 | 0.136 | 0.934 | 0.934 |
| Evenness (Simpson) | 0.001072 | 9.907 | **0.002** | **0.008** |
| Dominance (Berger-Parker) | - 0.0014 | 2.988 | 0.084 | 0.168 |
| **Stool** | | | | |
| Shannon diversity | 0.002489 | 2.2794 | 0.3199 | 0.6066667 |
| Genus richness (observed) | 0.1937 | 1.0823 | 0.2982 | 0.6066667 |
| Evenness (Simpson) | -0.0001366 | 0.5581 | 0.455 | 0.6066667 |
| Dominance (Berger-Parker) | 6.415e-05 | 0.0365 | 0.8484 | 0.8484000 |

**2. Supplementary methods**

**2.4. DNA-extraction** **and 16S sRNA gene sequencing, absolute quantification of bacterial load by qPCR**

DNA was extracted from the stool and sputum samples with the DNeasy Pow-

erSoil Pro Kit (QIAGEN, Germany). A bead-beating step of 30 herzt for 2 times 7 minutes on a TissueLyser (QIAGEN, Germany) was added to allow for a mechanical disruption of the cells in the sample. Prior to sputum DNA extraction, sputum was homogenized and extracellular human DNA was depleted with Benzonase (Merck, Germany) according to the Benzonase2 method (2).

The V4 region of the 16s rRNA genes was amplified as described in Klopp et al (3) using the primers 515f and 806r (forward: 5’-GTGCCAGCMGCCGCGGTAA-3’, reverse: 5’-GACTACHVGGGTWTCTAATCC-3’). Sequencing was performed on a MiSeq (Illumina, Inc., USA) for 251 cycles to produce paired-end reads. Negative controls were extracted, amplified and sequenced in tandem.

For all samples, qPCR was performed using an Applied Biosystems QuantStudio 3 system (Thermo Fisher Scientific, Darmstadt, Germany). Amplification and detection were performed in 96-well optical plates (Applied Biosystems) with SYBR-Green (Applied Biosystems). All amplifications were performed in duplicates in a final volume of 5 μL containing 13.8 μL of a 2xSYBR Green PCR Master Mix including ROX as a passive reference (Applied Biosystems), 500 nM of each primer (Univ 337 F 5′-ACTCCTACGGGAGGCAGCAGT-3′ and Univ 518 R 5′-GTATTACCGCGGCTGCTGGCAC-3′) and 1.2 μL of template DNA (0.5 µg/µL).

For amplification, the standard protocol of the Applied Biosystems QuantStudio 3 system was followed, i.e. an initial cycle at 95°C for 10 min, followed by 40 cycles at 95°C for 15 s, and 1 min at 60°C. To check for specificity, melting curve (Tm) analysis was performed, increasing the temperature from 60°C to 95°C at a rate of 0.2°C per second with the continuous monitoring of fluorescence.

Standard curves for quantification consisted of 10-fold serial dilutions in the range of 108–100 copies of the 16S rRNA gene of the E. coli (Invitrogen, C404010) amplified with primers 27 F (5′-GTTTGATCCTGGCTCAG-3′) and 1492 R (5′-CGGCTACCTTGTTACGAC-3′). Copy numbers per gram faeces were calculated for each primer set used.

**2.5. Bioinformatical and statistical analysis**

**16S sequence processing**

Sequences were pre-processed to infer amplicon sequence variants (ASVs) following the pipeline of DADA2 v1.18 (4). In brief, the raw forward and reverse reads (fastq) were truncated at base 245 and 240, respectively based on their quality score. Primer sequences were trimmed and the rest of the filtering parameters were kept with the default settings from the DADA2 pipeline. As an additional denoising step, fragments between 249 and 253 bp were selected *in silico* before removing chimeric sequences. The expected amplicon size was 251 bp. Taxonomic annotation was done following the naive Bayesian classifier from DADA2 with SILVA SSU database v138.1. Species were assigned just in case of exact matching of the 16S fragments. All ASVs, metadata and taxonomic information was compiled into a single object for further analysis using the package Phyloseq v1.22.3 (5).

**Data pre-processing**

We conducted data cleaning as follows: 1) removing samples with low read counts (less than 8000 reads), 2) discard unassigned or non-bacteria ASVs at phylum level, and 3) remove low prevalent ASVs that do not appear more than 5 times in more than 10% of the samples. Finally, we normalized the data by transforming ASV proportions by sample to an even depth (1E6). We split the data set based on their origin (stool and sputum) for further analysis

**Prediction of functional profiles**

The pre-processed 16S data was used to predict the functional profile from the two types of biosamples. For that aim, the PICRUSt2 package (Phylogenetic Investigation of Communities by Reconstruction of Unobserved States 2) makes accurate predictions about the functional capacity of a given community based on 16S data (6). PICRUSt2 provides the abundance of predicted metagenomes, genes and pathways in the bacterial community based on available bacterial whole genomes. PICRUSt2 interpolates a bacterial genome based on its nearest known taxonomic neighbor. The predictions were obtained following the pipeline described by the authors. In brief, the ASVs were aligned to reference sequences using HMMER (7) and placed into a reference tree using EPA-NG (8) and GAPPA (9). PICRUSt2 normalizes for multiple 16S gene copies in bacteria using the hidden state prediction tool, castor (10). The normalized data was used to predict gene family profiles and mapped onto gene pathways using MinPath (11).

**Alpha and beta diversity and estimation of dominant taxa**

To assess alpha diversity metrics, we used Chao1, Shannon, Pielou and Berger-Parker indices, these estimate richness, diversity, evenness and dominance, respectively. All three indices were calculated using the package Microbiome v1.13.8 (12). We compared alpha diversity between visits (Baseline V1, V2 and V3) for each sample type. Mann–Whitney U tests comparisons. We control for false discovery rate (FDR) employing the Benjamini-Hochberg procedure implemented with the package rstatix v0.7.0 (13). Beta diversity was assessed using Bray-Curtis dissimilarity index between samples and computed with vegan v2.5-7 (14). Comparisons of distance between individual patient microbiomes among visits were tested using Mann–Whitney U tests. We used Principal Coordinates Analysis (PCoA) with the vegan package v2.5-7 (14), for multivariate analysis with the Bray-Curtis distances. PERMANOVA tests for multivariate effect were done using the adonis function from the vegan package v2.5-7 (14) stratified for patient ID.

We identified the dominant taxa from each patient accounting for the highest relative abundance for all ASV at the genus level.

**Generalized linear mixed-effects models for statistical inference analyses**

Generalized linear mixed-effects models (GLMM) were calculated for different inference analyses using lme4 v1.1.26 (15) with patient ID as random factor. Inspections of residual plots did not reveal any deviations from homoscedasticity or normality. Pairwise comparisons for the interactions were calculated.

1) Considering lung function (ppFEV1) as response and alpha diversity (Shannon), visit and interaction, as explanatory variables; to test whether the faecal and sputum microbiome diversity could infer lung function (Supplementary Figures and Tables 5&6).

2) Considering Bray-Curtis dissimilarity within patients between visits as our proxy for microbial composition and the mean training frequency and time between visits as measurements of the sports intervention together with change in ppFEV1 and ppFVC as lung function parameters; to explore which parameter linked to the sports intervention and lung function might impact changes in microbial composition (Bray-Curtis dissimilarity). A forward and backward selection stepwise regression was computed using stepAIC() from MASS (16) to define the best model based on the Akaike information criterion (AIC). (Supplementary Figure 3 A&B, Supplementary Table 1&2)

3) Considering alpha diversity metrics and 16S copy number or Bray-Curtis dissimilarity to assess the impact of antibiotic burden and recent antibiotic intake with patients and visits as random factors.

GLMMs were compared through a likelihood ratio test implemented with lmtest v0.9.38 (17) (Supplementary Table 3&4).

**Multivariate correlation analysis**

To assess possible associations between ASV abundance and clinical characteristics, nutritional information, or medication we run a multivariate correlation analysis using MetadeconfoundR (18) as described in (19). First, we tested for correlations for each microbial feature with each covariate and use Spearman’s rho as standardized signed effect estimate. *P* values were FDR-adjusted with the BH method for each comparison of two data spaces, requiring FDR < 0.05 for significance. A post-hoc test was done to account for dependency between patient samples: for each of two correlated features, a mixed-effects model was fitted of the rank-transformed variable using the rank of the other as explanatory variable, with patient ID as a random effect. We report standardized non-parametric effect sizes (signed) Cliff’s delta metric.

**ANOVA with repeated measures for clinical parameters**

The evolution of clinical parameters was analyzed via a one-way ANOVA with repeated measures in IBM® SPSS®-Software Version 27. If Mauchly's Test of Sphericity indicated that the assumption of sphericity had been violated, a Greenhouse-Geisser correction was used.

**References for Supplementary Methods:**

1. Hillen B, Simon P, Schlotter S, Nitsche O, Bähner V, Poplawska K, et al. Feasibility and implementation of a personalized, web-based exercise intervention for people with cystic fibrosis for 1 year. BMC Sports Sci Med Rehabil [Internet]. 2021;13(1):1–11. Available from: https://doi.org/10.1186/s13102-021-00323-y

2. Nelson MT, Pope CE, Marsh RL, Wolter DJ, Weiss EJ, Hager KR, et al. Human and Extracellular DNA Depletion for Metagenomic Analysis of Complex Clinical Infection Samples Yields Optimized Viable Microbiome Profiles. Cell Rep [Internet]. 2019;26(8):2227-2240.e5. Available from: https://linkinghub.elsevier.com/retrieve/pii/S2211124719301287

3. Klopp J, Ferretti P, Meyer CU, Hilbert K, Haiß A, Marißen J, et al. Meconium Microbiome of Very Preterm Infants across Germany.

4. Callahan BJ, McMurdie PJ, Rosen MJ, Han AW, Johnson AJA, Holmes SP. DADA2: High-resolution sample inference from Illumina amplicon data. Nat Methods. 2016 Jun 29;13(7):581–3.

5. Mcmurdie PJ, Holmes S. phyloseq: An R Package for Reproducible Interactive Analysis and Graphics of Microbiome Census Data. Available from: www.plosone.org

6. Langille MGI, Zaneveld J, Caporaso JG, McDonald D, Knights D, Reyes JA, et al. Predictive functional profiling of microbial communities using 16S rRNA marker gene sequences. Nat Biotechnol [Internet]. 2013;31(9):814–21. Available from: http://dx.doi.org/10.1038/nbt.2676

7. Eddy SR. Accelerated Profile HMM Searches. Cit Eddy SR [Internet]. 2011;7(10):1002195. Available from: www.ploscompbiol.org

8. Barbera P, Kozlov AM, Czech L, Morel B, Darriba D, Flouri T, et al. EPA-ng: Massively Parallel Evolutionary Placement of Genetic Sequences. Syst Biol. 2019;68(2):365–9.

9. Czech L, Barbera P, Stamatakis A. Genesis and Gappa: Processing, analyzing and visualizing phylogenetic (placement) data. Bioinformatics. 2020;36(10):3263–5.

10. Louca S, Doebeli M. Efficient comparative phylogenetics on large trees. Bioinformatics. 2018;34(6):1053–5.

11. Ye Y, Doak TG. A Parsimony Approach to Biological Pathway Reconstruction/Inference

for Genomes and Metagenomes. PLoS Comput Biol [Internet]. 2009;5(8):1000465. Available from: www.ploscompbiol.org

12. Lahti L, Shetty S. microbiome R package [Internet]. Available from: http://microbiome.github.io

13. Kassambara A. rstatix. 2021.

14. Oksanen J, Blanchet FG, Friendly M, Kindt R, Legendre P, McGlinn D, et al. vegan: Community Ecology Package [Internet]. Available from: https://cran.r-project.org/package=vegan

15. Bates D, Mächler M, Bolker BM, Walker SC. Fitting linear mixed-effects models using lme4. J Stat Softw. 2015;67(1).

16. Venables W, Ripley B. Modern Applied Statistics with S [Internet]. Fourth. New York: Springer US; 2002. Available from: https://www.stats.ox.ac.uk/pub/MASS4/

17. Zeileis A, Hothorn T. Diagnostic Checking in Regression Relationships. R News [Internet]. 2002;2(3):7–10. Available from: http://cran.r-project.org/doc/Rnews/

18. Birkner T. metadeconfoundR [Internet]. 2021. Available from: https://github.com/TillBirkner/metadeconfoundR

19. Balogh A, Bartolomaeus H, Löber U, Avery EG, Steckhan N, Markó L, et al. Fasting alters the gut microbiome with sustained blood pressure and body weight reduction in metabolic syndrome patients. Nat Commun. 2020;
